# Searching for health beneficial *n*-3 and *n*-6 fatty acids in plant seeds

**DOI:** 10.1002/ejlt.201100008

**Published:** 2012-02

**Authors:** Katrin Kuhnt, Christian Degen, Anke Jaudszus, Gerhard Jahreis

**Affiliations:** Institute of Nutrition, Department of Nutritional Physiology, Friedrich Schiller UniversityJena, Germany

**Keywords:** α-linolenic acid, γ-linolenic acid, Boraginaceae, stearidonic acid, vegetable *n*-3 PUFA

## Abstract

Various plant seeds have received little attention in fatty acid research. Seeds from 30 species mainly of Boraginaceae and Primulaceae were analysed in order to identify potential new sources of the *n*-3 PUFA α-linolenic acid (ALA) and stearidonic acid (SDA) and of the *n*-6 PUFA γ-linolenic acid (GLA). The fatty acid distribution differed enormously between genera of the same family. *Echium* species (Boraginaceae) contained the highest amount of total *n*-3 PUFA (47.1%), predominantly ALA (36.6%) and SDA (10.5%) combined with high GLA (10.2%). Further species of Boraginaceae rich in both SDA and GLA were *Omphalodes linifolia* (8.4, 17.2%, resp.), *Cerinthe minor* (7.5, 9.9%, resp.) and *Buglossoides purpureocaerulea* (6.1, 16.6%, resp.). *Alkanna* species belonging to Boraginaceae had comparable amounts of ALA (37.3%) and GLA (11.4%) like *Echium* but lower SDA contents (3.7%). Different genera of Primulaceae (*Dodecatheon* and *Primula*) had varying ALA (14.8, 28.8%, resp.) and GLA portions (4.1, 1.5%, resp.), but similar amounts of SDA (4.9, 4.5%, resp.). *Cannabis sativa* cultivars (Cannabaceae) were rich in linoleic acid (57.1%), but poor in SDA and GLA (0.8, 2.7%, resp.). In conclusion, several of the presented plant seeds contain considerable amounts of *n*-3 PUFA and GLA, which could be relevant for nutritional purposes due to their biological function as precursors for eicosanoid synthesis.

**Practical applications:**

*N*-3 PUFA are important for human health and nutrition. Unfortunately, due to the increasing world population, overfishing of the seas and generally low amounts of *n*-3 PUFA in major oil crops, there is a demand for new sources of *n*-3 PUFA. One approach involves searching for potential vegetable sources of *n*-3 PUFA; especially those rich in ALA and SDA. The conversion of ALA to SDA in humans is dependent on the rate-limiting Δ6-desaturation. Plant-derived SDA is therefore a promising precursor regarding the endogenous synthesis of *n*-3 long-chain PUFA in humans. The present study shows that, in addition to seed oil of *Echium*, other species of Boraginaceae (*Cerinthe, Omphalodes, Lithospermum, Buglossoides*) and Primulaceae (*Dodecatheon, Primula*), generally high in *n*-3 PUFA (30–50%), contain considerable amounts of SDA (5–10%). Therefore, these seed oils could be important for nutrition.

## Introduction

The *n*-3 and *n*-6 polyunsaturated fatty acids (PUFA) are essential for normal human growth and development and further appear to be important for the prevention and treatment of several diseases (coronary heart disease, arthritis, inflammatory disorders). They are involved in the eicosanoid synthesis and are components of cellular membranes 1–3.

Higher plants are able to produce the essential *n*-6 and *n*-3 PUFA: linoleic acid (LNA; C18:2*c*9,12) and α-linolenic acid (ALA; C18:3*c*9,12,15), respectively. In contrast, animals and humans have no capacity to synthesize LNA and ALA from the precursor oleic acid (C18:1*c*9) due to the lack of Δ12- and Δ15-desaturases. LNA and ALA are substrates for endogenous elongation and desaturation processes and share the same enzyme systems (e.g., Δ6- and Δ5-desaturases, elongases) to produce long-chain PUFA (LC-PUFA) such as arachidonic acid (AA; C20:4*c*5,8,11,14) and eicosapentaenoic acid (EPA; C20:5*c*5,8,11,14,17), respectively.

Modern Western diets are not only low in *n*-3 PUFA but also too high in *n*-6 PUFA, deriving mainly from meat and vegetable oils rich in LNA (corn, sunflower and safflower) 1. Thus, nutritional pro- and anti-inflammatory eicosanoid precursors become imbalanced. The main dietary source of EPA and docosahexaenoic acid (DHA; C22:6*c*4,7,10,13,16,19) is marine fish fed on marine microbes (micro algae, diatoms, bacteria). By themselves, mammals and marine fish have a very limited capacity to produce the important *n*-3 LC-PUFA.

Due to the increasing world population and the overfishing of the seas the supply of *n*-3 LC-PUFA is limited. Several work groups pursue strategies to develop alternative sources of *n*-3 PUFA such as transgenic plants, aquaculture of marine microbes and fish, and searching for plant oils rich in *n-*3 PUFA 4, 5. There are only low amounts of *n*-3 PUFA in major oil crops such as soybean, sunflower and palm oil 6, 7. The PUFA synthesis occurs in all plant cells, and therefore PUFA are present in the leaves and roots, but mainly in seeds in varying amounts 8. Several studies examined Boraginaceae species as relevant sources of health beneficial *n*-3 and *n*-6 fatty acids 9–12.

ALA is the most abundant *n*-3 PUFA in plant oils, such as linseed oil 7, 13. In the human body, the endogenous synthesis of *n*-3 LC-PUFA by elongation and desaturation of ALA to EPA and particularly to DHA is limited 14. A further interesting *n*-3 PUFA is stearidonic acid (SDA; C18:4*c*6,9,12,15). SDA is present only in species of few plant families, for example Boraginaceae 15. Plant-derived SDA is a promising precursor regarding endogenous synthesis of *n*-3 LC-PUFA in humans, because the rate-limiting Δ6-desaturation of ALA to SDA already occurred effectively in the plant.

The γ-linolenic acid (GLA; C18:3*c*6,9,12) is also a Δ6-desaturation product but of LNA and is found primarily in vegetable oils. High levels of GLA are found mainly in Boraginaceae and in other families such as Ranunculaceae and Primulaceae 12, 16. GLA is described as a fatty acid with anti-inflammatory properties 17. However, its therapeutic value is controversially discussed (atopic dermatitis, rheumatoid arthritis) 17.

To improve the supply with *n*-3 LC-PUFA for human nutrition new vegetable oil sources containing precursors, such as SDA, have to be identified. In this work, the fatty acid composition of seeds from various species of plant families potentially high in ALA, SDA and GLA, such as Primulaceae and Boraginaceae, were investigated.

## Materials and methods

### Seed classification

The seeds were provided by different botanical gardens and JPR Natural Products (Germany). In total, 30 species of four families (Primulaceae, Boraginaceae, Elaeagnaceae, Cannabaceae; Table 1) were analysed. Boraginaceae consisted of eleven different genera (Table 2). A total of six cultivars of *Cannabis sativa* (Bialo-brzeskie, Beniko, Futura, Fedora 17, Felina 32, Chameleon) from three countries (Poland, The Netherlands, France) were investigated. For *B. purpureocaerulea (L.) I.M. Johnst.* a different spelling was found to be possible (*B. purpurocaerulea*) and this species is also known as *Lithospermum purpurocaeruleum* (*L.*) 9.

**Table 1:** Fatty acid distribution of seeds of different plant families (% of total FAME)

Family		Elaeagnaceae†		Cannabaceae‡		Primulaceae		Boraginaceae	
					
Different genera		*n* = 1		*n* = 1		*n* = 2		*n* = 11	
Different species§		*n* = 1		*n* = 1 (×6#)		*n* = 7		*n* = 16	
					
		Mean	SD	Mean	SD	Mean	SD	Mean	SD
	Oil content	1.7^d^	0.2	18.6^a^	1.3	12.9^c^	2.7	15.3^b^	5.8
	Fatty acid (% of total FAME)									
	C16:0	9.63	0.21	6.69	0.39	7.98	0.75	8.90	7.17
	C16:1*c*9	2.35^a^	0.04	0.12^c^	0.03	0.49^b^	0.08	0.05^d^	0.06
	C18:0	3.03^a^	0.04	2.85^a^	0.26	1.15^b^	0.22	2.81^a^	1.24
*n*-9	C18:1*c*9	19.4^a^	0.22	11.4^b^	1.15	25.6^a^	6.19	22.2^a^	10.5
	C18:1*c*11	2.40^a^	0.02	0.85^c^	0.04	1.42^b^	0.11	0.67^c^	0.36
*n*-6	C18:2*c*9,12	39.8^b^	0.13	57.1^a^	0.53	33.1^c^	2.64	22.3^d^	4.59
*n*-6	C18:3*c*6,9,12	1.11^b^	0.01	2.71^b^	0.59	2.95^b^	1.43	9.67^a^	4.04
*n*-3	C18:3*c*9,12,15	20.0	0.04	15.6	0.67	20.8	7.75	21.6	13.7
*n*-3	C18:4*c*6,9,12,15	0.36^b^	0.01	0.77^b^	0.13	4.73^a^	0.62	4.99^a^	3.41
	C20:0	0.66^a^	0.01	0.87^a^	0.04	0.15^c^	0.07	0.25^b^	0.25
	C20:1*c*11	0.40^b^	0.03	0.50^b^	0.14	0.21^b^	0.10	2.64^a^	2.01
	C22:0	0.28	0.28	0.34	0.34	0.12	0.12	0.24	0.24
	C22:1*c*13	0.00^b^	0.00	0.00^b^	0.00	0.04^b^	0.11	2.58^a^	3.76
	C24:0	0.00	0.00	0.09	0.06	0.07	0.11	0.02	0.05
	C24:1*c*15	0.00^b^	0.00	0.00^b^	0.00	0.00^b^	0.02	0.86^a^	1.10
	Σ unidentified FA	0.08	0.16	0.04	0.10	0.27	0.30	0.09	0.11
									
	Σ SFA	14.1	0.12	10.9	0.57	9.59	0.89	12.3	8.31
	Σ MUFA	24.5^a^	0.25	12.9^b^	1.1	27.8^a^	6.45	29.1^a^	16.6
	Σ PUFA	61.3^b^	0.09	76.2^a^	1.27	61.7^b^	5.67	58.6^b^	19.7
	Σ *n*-3	20.3	0.05	16.4	0.66	25.6	7.63	26.6	16.5
	Σ *n*-6	40.9^b^	0.12	59.8^a^	0.91	36.0^c^	3.49	32.0^c^	7.01
	*n-*6/*n*-3	2.01^b^	0.01	3.66^a^	0.14	1.53^b^	0.49	2.11^b^	1.88

† Hippophae, Hippophae rhamnoides L.

‡ Cannabis, Cannabis sativa L.

§ Four samples per species were analysed.

# Includes six cultivars.

^a,b,c,d^ Means with different superscript letters indicate differences between plant families (^a^ highest value of a family, post-hoc Scheffé-test, *P* < 0.05).

**Table 2:** Oil content (%) and fatty acid distribution (% of total FAME) of seeds of different genera and species of Primulaceae and Boraginaceae [to be continued]

	Family	Primulaceae								
		
	Genus	*Dodecatheon*				***D.*** **spp. *mean***	*Primula*			***P.*** **spp. *mean***
					
	Species	*D. pulchellum (Raf.) Merr.*	*D. clevelandii Greene*	*D. meadia L.*	*D. jeffreyi Van Houtte*		*P. veris L.*	*P. ruprechtii Kusn.*	*P. denticulata Sm.*	
	Samples	*n* = 4	*n* = 4	*n* = 4	*n* = 4	*n* = 16	*n* = 4	*n* = 4	*n* = 4	*n* = 12
	Oil content (%)	13.9	8.87	19.9^3^	11.2	**13.5**^**d**^	11.1	14.8	16.8	**14.2**^**d**^
	Fatty acid (% Σ FAME)										
	C16:0	8.50	7.47	7.47	8.09	**7.88**^**a**^	8.12	7.29	8.95	**8.12**^**a**^
	C16:1*c*9	0.64^1^	0.51^2^	0.47^2^	0.49^2^	**0.53**^**a**^	0.50^2^	0.45^2^	0.39^3^	**0.45**^**b**^
	C17*iso*	0.27	0.36^3^	0.31	0.45^2^	**0.35**^**b**^	0.28	0.24	1.17^1^	**0.56**^**a**^
	C18:0	1.22	0.98	1.05	0.98	**1.06**^**c**^	1.48	1.22	1.08	**1.26**^**b**^
*n*-9	C18:1*c*9	29.8^3^	30.7^3^	31.9^3^	29.2	**30.4**^**b**^	22.0	18.8	16.6	**19.2**^**c**^
	C18:1*c*11	1.49^2^	1.48^2^	1.42^2^	1.46^2^	**1.46**^**a**^	1.42^2^	1.51^2^	1.18^3^	**1.37**^**b**^
*n*-6	C18:2*c*9,12	33.3^2,3^	33.3^2,3^	32.2^3^	35.5^1,2^	**33.6**^**a**^	30.3	30.2	36.8^1^	**32.4**^**a**^
*n*-6	C18:3*c*6,9,12	3.84	4.04	3.94	4.38	**4.05**^**d**^	1.86	1.04	1.57	**1.49**^**e**^
*n*-3	C18:3*c*9,12,15	15.0	14.8	15.4	14.1	**14.8**^**c**^	27.2	31.2	28.0	**28.8**^**b**^
*n*-3	C18:4*c*6,9,12,15	4.88	5.04	5.15	4.57	**4.91**^**b**^	4.42	5.52	3.56	**4.50**^**c**^
	C20:0	0.08	0.14	0.12	0.10	**0.11**^**c**^	0.25	0.20	0.15	**0.20**^**c**^
	C20:1*c*11	0.22	0.41	0.22	0.20	**0.26**^**c**^	0.17	0.14	0.09	**0.14**^**d**^
	C22:0	0.00	0.13	0.07	0.08	**0.07**^**c**^	0.23	0.22	0.09	**0.18**^**b**^
	C22:1*c*13	0.00	0.30	0.00	0.00	**0.08**^**c**^	0.00	0.00	0.00	**0.00**^**d**^
	C24:0	0.00	0.00	0.00	0.04	**0.01**^**b**^	0.20^2^	0.23^1^	0.03	**0.15**^**a**^
	C24:1*c*15	0.00	0.03	0.00	0.00	**0.01**^**b**^	0.00	0.00	0.00	**0.00**^**b**^
										
	Σ SFA	10.3	8.82	8.76	9.39	**9.32**^**b**^	10.3	9.21	10.3	**9.96**^**a**^
	Σ MUFA	32.3	33.5	34.1	31.4	**32.8**^**b**^	24.2	20.9	18.4	**21.1**^**c**^
	Σ PUFA	57.0	57.3	56.7	58.6	**57.4**^**c**^	64.1	68.4	70.0^3^	**67.5**^**b**^
	Σ *n*-3	19.9	19.9	20.5	18.7	**19.7**^**d**^	31.6	36.7	31.6	**33.3**^**c**^
	Σ *n*-6	37.1^3^	37.3^2,3^	36.1^3^	39.9^2,3^	**37.6**^**a**^	32.2	31.2	38.4^2,3^	**33.9**^**b,c**^
	Σ *n*-6/*n*-3	1.87	1.88	1.76	2.14	**1.91**^**b**^	1.02	0.85	1.22	**1.03**^**c**^

	Family	Boraginaceae																		
		
	Genus	*Echium*			***E. species*** **mean**	*Alkanna*		***A. species*** **mean**	*Cynoglossum*			***C. species*** **mean**	*Various genera*								
								
													*Moltkia*	*Myosotis*	*Arnebia*	*Cerinthe*	*Ompha-lodes*	*Anchusa*	*Litho-spermum*	*Buglossoides*
	Species	*E. vulgare L. (1)*	*E. vulgare L. (2)*	*E. rosulatum Lange*		*A. graeca Boiss. & Spruner*	*A. orientalis (L.) Boiss.*		*C. officinale L. (1)*	*C. officinale L. (2)*	*C. germani-cum Jacq.*		*M. suffruticosa (L.) Brand*	*M. palustris (L.) Nath.*	*A. pulchra Edm.*	*C. minor L.*	*O. linifolia (L.) Moench*	*A. officinalis L.*	*L. officinale L.*	*B. purpureo-caerulea (L.) I.M. Johnst.*§
			
	Samples	*n* = 4	*n* = 4	*n* = 4	*n* = 12	*n* = 4	*n* = 4	*n* = 8	*n* = 4	*n* = 4	*n* = 4	*n* = 12	*n* = 4	*n* = 4	*n* = 4	*n* = 4	*n* = 4	*n* = 4	*n* = 4	*n* = 4
	Oil content (%)	16.7	13.8	20.6^3^	**17.0**^**c**^	23.1^2^	22.1^2^	**22.6**^**a**^	15.6	19.4^3^	24.3^1^	**19.8**^**b**^	13.8	14.9	3.32	11.5	16.5	12.7	10.6	6.15
	Fatty acid [% Σ FAME]																				
	C16:0	5.78	6.26	5.88	**5.97**^**c**^	4.97	4.93	**4.95**^**d**^	5.91	5.87	8.19	**6.65**^**b**^	10.43	9.17	32.1†	6.97	6.56	10.50	9.61	9.19
	C16:1	0.00	0.02	0.09	**0.03**^**c**^	0.01	0.00	**0.01**^**c**^	0.02	0.00	0.14	**0.05**^**c**^	0.10	0.16	0.04	0.00	0.00	0.06	0.04	0.05
	C17*iso*	0.00	0.00	0.00	**0.00**^**c**^	0.00	0.00	**0.00**^**c**^	0.00	0.00	0.00	**0.00**^**c**^	0.00	0.00	0.00	0.00	0.00	0.00	0.00	0.00
	C18:0	2.66^2,3^	2.86^2,3^	2.42^2,3^	**2.64**^**a**^	2.03^3^	2.26^2^	**2.14**^**a**^	1.28	1.52	1.45	**1.42**^**b**^	3.49^2,3^	3.75^2^	6.34^1^	3.39^2,3^	3.45^2,3^	2.11^3^	3.15^2,3^	2.78^2,3^
*n*-9	C18:1*c*9	12.3	13.0	12.0	**12.4**^**e**^	13.0	15.4	**14.2**^**d**^	30.6^3^	35.2^2^	38.4^1^	**34.7**^**a**^	38.1^1^	37.1^1^	27.6	17.1	10.3	24.2	17.6	13.1^b^
	C18:1*c*11	0.46	0.49	0.62	**0.52**^**d**^	0.42	0.46	**0.44**^**e**^	0.49	0.58	0.83	**0.63**^**c**^	0.50	0.72	1.72^1^	0.49	0.38	0.72	1.12^3^	0.77^b^
*n*-6	C18:2*c*9,12	21.0	20.6	18.6	**20.0**^**c**^	24.1	24.7	**24.4**^**b**^	30.8^3^	26.2	19.2	**25.4**^**b**^	17.1	25.2	13.8	22.4	26.2	27.6	19.1	20.7^b^
*n*-6	C18:3*c*6,9,12	11.6	10.5	8.51	**10.2**^**b**^	12.1	10.8	**11.4**^**a**^	8.17	6.59	2.41	**5.72**^**c**^	5.67	6.35	4.04	9.94	17.3^1^	11.1	13.1^3^	16.6^2^
*n*-3	C18:3*c*9,12,15	34.4	34.7	40.9^1^	**36.6**^**a**^	38.1^2^	36.5^3^	**37.3**^**a**^	3.36	5.15	6.72	**5.08**^**d**^	5.46	8.10	6.53	29.7	25.4	13.1	28.4	28.6
*n*-3	C18:4*c*6,9,12,15	11.1^1^	10.6^2^	9.86^3^	**10.5**^**a**^	3.91	3.46	**3.69**^**d**^	1.61	2.09	1.26	**1.66**^**e**^	1.95	2.19	1.67	7.51	8.44	2.64	5.42	6.14
	C20:0	0.00	0.02	0.03	**0.02**^**d**^	0.05	0.04	**0.04**^**d**^	0.55^2^	0.47^3^	0.31	**0.44**^**a**^	0.82^1^	0.46^3^	0.56^2^	0.17	0.15	0.25	0.00	0.12
	C20:1*c*11	0.68	0.68	0.65	**0.67**^**b**^	0.76	0.89	**0.82**^**b**^	5.66^2^	6.07^1^	4.89	**5.54**^**a**^	5.26^3^	4.29	3.20	1.32	1.08	3.96	1.71	1.08
	C22:0	0.00	0.00	0.00	**0.00**^**d**^	0.01	0.00	**0.01**^**d**^	0.68^2^	0.52^3^	0.45	**0.55**^**a**^	1.37^1^	0.24	0.06	0.15	0.03	0.27	0.00	0.02
	C22:1*c*13	0.00	0.10	0.10	**0.07**^**c**^	0.11	0.16	**0.13**^**b**^	8.08^2^	7.03^3^	12.2^1^	**9.10**^**a**^	7.45	1.05	1.04	0.50	0.32	2.71	0.34	0.18
	C24:0	0.00	0.00	0.00	**0.00**^**b**^	0.00	0.00	**0.00**^**b**^	0.09	0.00	0.00	**0.03**^**b**^	0.13^3^	0.03	0.03	0.00	0.00	0.00	0.00	0.07
	C24:1*c*15	0.00	0.01	0.04	**0.02**^**b**^	0.02	0.04	**0.03**^**b**^	2.47^3^	2.68^2^	3.56^1^	**2.90**^**a**^	1.61	0.71	1.12	0.27	0.23	0.59	0.30	0.14
																				
	Σ SFA	8.50	9.25	8.42	**8.72**^**b**^	7.09	7.23	**7.16**^**c**^	8.50	8.38	10.40	**9.09**^**b**^	16.41^2^	14.14^3^	39.26^1^	10.67	10.19	13.12	12.76	12.30
	Σ MUFA	13.4	14.3	13.5	**13.8**^**e**^	14.5	17.2	**15.9**^**d**^	47.3^3^	51.5^2^	60.0^1^	**53.0**^**a**^	53.1^2^	44.0	34.7	19.9	12.3	32.3	21.3	15.5
	Σ PUFA	78.0^1^	76.3^1^	77.8^1^	**77.4**^**a**^	78.2^1^	75.5^2,3^	**76.9**^**a**^	44.0	40.1	29.6	**37.9**^**d**^	30.1	41.8	26.0	69.5	77.5^1^	54.6	66.0	72.1^3^
	Σ *n*-3	45.5^2^	45.2^2^	50.7^1^	**47.1**^**a**^	42.0^3^	40.0	**41.0**^**b**^	4.98	7.25	7.98	**6.77**^**e**^	7.41	10.30	8.20	37.2	33.8	15.8	33.8	34.8
	Σ *n*-6	32.5	31.1	27.1	**30.2**^**d**^	36.2^3^	35.5	**35.9**^**a,b**^	39.1^2,3^	32.8	21.6	**31.2**^**d**^	22.7	31.5	17.8	32.3	43.7^1^	38.8^2,3^	32.2	37.3^2,3^
	*n*-6/*n*-3	0.71	0.69	0.53	**0.65**^**d**^	0.86	0.89	**0.88**^**d**^	7.85^1^	4.53^2^	2.71	**5.03**^**a**^	3.07	3.06	2.17	0.87	1.29	2.46	0.95	1.07

^a,b,c,d,e^ Means with different superscript letters indicate differences between genera (^a^ highest value of a genus, post-hoc Scheffé-test, *P* < 0.05).

^1,2,3^ Means with different superscript numbers indicate differences between species (^1^ highest value of a species, post-hoc Scheffé-test *P* < 0.05; for a better overview the comparison between different species is given only up to the third ^[3]^ highest value).

† Significantly highest value compared to all other values in this row.

§ B. purpureocaerulea L. synonym Lithospermum purpurocaeruleum L.

### Lipid extraction

The dried seeds were finely ground and homogenised with a laboratory mill for small amounts. Total lipids were extracted from 1.0 g samples (or lower) with methanol/chloroform/water (2:1:1 by volume). The lipid extracts were transesterified with trimethylsulphonium hydroxide (TMSH; 5 mg lipid extract; 200 µL methanol, SeccoSolv® MERCK; 100 µL TMSH solution, MACHEREY-NAGEL; Germany, incubation with TMSH at 80°C for 15 min) to produce FAME directly within the injector of the GC at 260°C.

### Fatty acid analysis

The analysis of FAME extracts was performed by GC (GC-17 V3, Shimadzu, Japan) equipped with a cooled autosampler and FID. A fused-silica capillary column of medium polarity was used (DB-225MS: 60 m × 0.25 mm id with a 0.25 µm film thickness; Agilent Technologies, US). The initial oven temperature was maintained at 70°C for 2 min, then increased by 10°C/min to 180°C, then increased by 2°C/min to 220°C and held for 5 min. Finally, it was increased by 2°C/min to 230°C and held for 15 min. The injector and detector temperatures were maintained at 260°C. Hydrogen was used as carrier gas 18.

### Identification and calculation of FAME

The total lipid content of the seeds was expressed as % of the air-dried seeds. The amount of the separated FAME was expressed as % of the total FAME. Various reference standards were used as FAME mix to identify fatty acid peaks: No. 463, 674, (NU-CHEK PREP; INC., US), BR2, BR4, ME 93 (Larodan; Sweden), Supelco® 37 Component FAME Mix, PUFA No. 3. LabSolutions software for GC (GCsolution; Shimadzu, Japan) was used for peak integration.

### Statistics

All statistical analyses were performed using SPSS statistics, version 17.0 (©2009 SPSS Statistics 17 Inc, Illinois, US) with *P*≤0.05 to indicate significant differences. The means of each species resulted from quadruplicates. The SD is given only for the comparison between the families. The difference of means between families, genera and species was tested with the multivariate post-hoc Scheffé-test.

## Results

The total oil content of the 30 analysed species varied from 1.7% in *Hippophae rhamnoides* (Table 1) to 24.3% in *Cynoglossum germanicum* (Table 2). The mean oil content was 15.0 ± 5.4%.

### Comparison between families

The mean saturated fatty acids (SFA), total *n*-3 PUFA and ALA contents did not differ between the families. However, Primulaceae and Boraginaceae were the families with the highest mean SDA amounts (4.7 and 5.0%, resp.). Additionally, Boraginaceae were richest in GLA (9.7%). The analysis of Cannabaceae included only *Cannabis sativa* cultivars which showed the lowest MUFA contents (12.9%) but the highest PUFA contents (76.2%) with mainly LNA (57.1%; Table 1). Among Elaeagnaceae, only *Hippophae rhamnoides* seeds were analysed, which showed the highest palmitoleic acid (C16:1*c*9) and *cis*-vaccenic acid (C18:1*c*11) contents (2.4, 2.4%, resp.). In general, no EPA and DHA could be detected in the seed oils.

### Comparison between genera

Genera, of which only one species was analysed (*Hippophae, Cannabis* and various genera of Boraginaceae), were excluded from the genus comparison. Interestingly, separated into single genera of the same family, several fatty acids differed enormously, particularly GLA, ALA and SDA contents (Table 1). Genera of Primulaceae *Dodecatheeon* and *Primula* showed moderate SDA amounts of 4.9 and 4.5%, respectively. However, *Primula* species had a low GLA content (1.5%) but a high ALA content (28.8%). *Echium* and *Alkanna* species (Boraginaceae) contained the highest GLA amount (10.2, 11.4%, resp.) with a simultaneously high ALA content (mean 36.6%). The distinctiveness of *Echium* species was their additionally highest SDA amount (10.5%). Therefore, among all genera, *Echium* had the highest *n*-3 PUFA content (47.1%). *Cynoglossum* (Boraginaceae) contained the highest MUFA level (53.0%; mainly oleic acid 34.7%). Furthermore, *Cynoglossum* species contained the highest amounts of *n-*9 very LC (VLC)-MUFA, such as eicosenoic acid (C20:1*c*11, 5.5%), erucic acid (C22:1*c*13, 9.1%) and nervonic acid (C24:1*c*15, 2.9%; Table 2).

### Comparison between species

Among all the analysed species, *E. rosulatum* showed the highest *n*-3 PUFA content resulting from high ALA and SDA contents (40.9 and 9.9%, resp.), and the lowest *n*-6/*n*-3 ratio (0.5; Table 2). *E. vulgare* had significantly higher SDA contents (11.1, 10.6%) compared to *E. rosulatum* (9.9%; Table 2). Furthermore, species of Boraginaceae such as *Omphalodes linifolia* and *Buglossoides purpureocaerulea* were characterised by extraordinarily high GLA (17.3, 16.6%, resp.) and high SDA amounts (8.4, 6.1%, resp.). In addition, both species contained high ALA portions (25.4, 28.6%, resp.; Table 2). Appreciable SDA and GLA contents were also found in *Cerinthe minor* (7.5, 5.4%, resp.) and *Lithospermum officinale* (9.9, 13.1%, resp.).

*Arnebia pulchra* was the only species containing a plant atypically high SFA amount (39.3%), with mainly C16:0 (32.1%). *Cynoglossum germanicum* was the species with the highest *n*-9 VLC-MUFA content characterised by erucic acid with 12.2% and nervonic acid with 3.6%. *Moltkia suffruticosa* had the highest C22:0 content (1.4%) and showed also high *n*-9 VLC-MUFA contents (C20:1*c*11, 5.3%; C22:1*c*13, 7.5%; C24:1*c*15, 1.6%; Table 2). The branched-chain fatty acid C17:0*iso* was comparatively high in species of Primulaceae and highest in *P. denticulata* (1.2%).

## Discussion

*Echium* encompasses over 50 species which belong to Boraginaceae. Many *Echium* species showed high *n*-3 PUFA contents 12, 15. The present study, aiming at detecting potential *n*-3 PUFA plant sources (Table 2), shows that *Echium* species stand out by their unique composition of high contents of SDA (10.5%) and ALA (36.6%) together with a high content of GLA (10.2%). *E. vulgare* (*1*) showed the highest SDA content (11.1%) in combination with high amounts of ALA (34.4%) and GLA (11.6%; Table 2). Further fatty acid analyses of *E. vulgare* showed similarly high amounts of SDA (9.7, 9.9%), ALA (34.1, 33.9%) and GLA (11.7, 9.2%), respectively 11, 16. The SDA portion in *E. vulgare* seed oil is critically dependent on the presence of the Δ6-desaturation of ALA while the SDA synthesis via Δ15-desaturation of GLA is highly unlikely 19. *Echium humile* ssp. *Pycnanthum* (Pomel) Greuter & Burdet 2 and *E. asperrimum* have been shown to contain highest SDA with 16.2% 12 and 21.1% 16, respectively. However, not all *Echium* species are rich in SDA (*E. flavum* 3.1%) 16.

From a nutritional point of view, Echium oil could increase the *n*-3 PUFA intake in humans. In addition, it has been shown that highly concentrated SDA was converted to EPA more efficiently than ALA in humans 20. Application of natural Echium oil effectively results in an enrichment of EPA in human tissues (21, 22; unpublished results 23). *E. plantagineum* was established as a SDA*-*rich *Echium* species 12. In 2007, the US Food & Drug Administration declared refined Echium oil as novel food for the market and the oil from *E. plantagineum* is available as food ingredient (e.g., INCROMEGA V3 with 12.5% SDA; Croda GmbH, Germany). Furthermore, a new biotechnologically-derived soybean oil with 20% SDA was created (SDA-20; Monsanto Company, MO, US) 24.

In the present work, beside the *Echium* species, further SDA-containing species of Boraginaceae were found such as *Omphalodes linifolia* 8.4%>*Cerinthe minor* 7.5%>*Buglossoides purpureocaerulea* 6.1%>*Lithospermum officinale* 5.4% (Table 2).

To our knowledge, the only report on the fatty acid profile of *Omphalodes linifolia* 11 to date shows a much lower percentage of SDA (3.5%) as well as of ALA (8.8%) than presented herein (Table 2). However, in *Cerinthe minor* we detected similar SDA and ALA portions (7.3, 29.0%, resp.; 11) (Table 2). Although the closely related *C. major* was not subject of our analyses, it is worth mentioning that despite high ALA, it contains only marginal amounts of SDA 11. Well corresponding to our data, *Buglossoides purpureocaerulea* (syn. *Lithospermum purpurocaeruleum*) was previously been found to be a relevant source of SDA with 7% 9. Among *Lithospermum* species, *L. arvense* has been found to be relatively rich in SDA with a portion up to 17% 9, 11. For seeds of *Lithospermum officinale*, varying levels of SDA ranging from 7.0 to 13.3% have been reported 11 that were generally higher than the present finding (5.4%; Table 2). In general, *Echium* species were formerly and herein confirmed to be good sources of SDA (12, Table 2).

In addition to high SDA portions, we detected comparably high GLA accounting for 17% in both *Omphalodes linifolia* and *Buglossoides purpureocaerulea* (Table 2). Also, the analysed *Echium* species as well as *Alkanna graeca* and *orientalis*, *Anchusa officinalis* and *Lithospermum officinale* were relatively rich in GLA with about 10% 11 (Table 2).

Seed oils known to be rich in GLA are, e.g., borage oil (*Borago officinalis* 17–28%), evening primrose oil (*Oenothera biennis* 7–10%) and black current seed oil (*Ribes nigrum* 10–13%) 6, 11, 25. In comparison, in seed oils from *Cannabis sativa* (hempseed oil) we found comparably low GLA amounts (1.8–3.6%; Table 1). However, the improvement of clinical symptoms in patients with atopic dermatitis was attributed to the high total PUFA (70–80%; Table 1) in orally applied hempseed oil 26.

SDA and GLA are Δ6-desaturation products derived from ALA and LNA, respectively. Thus, SDA and GLA appear jointly in few seed oils such as of *Echium* species with a unique SDA to GLA ratio of 1:1 (Table 2), most likely due to high activity of the Δ6-desaturase 19. The Δ6-desaturase is classified as an acyl-lipid desaturase that inserts a further double bond between the pre-existing double bond and the carboxyl end of the fatty acid. Its expression strongly depends on tissue and plant species 13. New data show that Δ6-desaturases of different species, even of the same genus (e.g., *Primula*), have different substrate preferences, for example, the Δ6-desaturase with *n*-3 selectivity 27, 28. Consequently, in the analysed *Primula* species the SDA portion was considerably higher than the GLA portion (5:2; Table 2), despite similar LNA and ALA portions.

The branched-chain fatty acid C17:0*iso* found in *P. denticulata* could have been originated from surface waxes. However, no respective data were available.

The oils from *Moltkia suffructicosa and Cynoglossum* species were found to be relatively rich in *n*-9 VLC-MUFA, such as erucic acid with 7.5 and 9.1%, respectively (Table 2). Erucic acid is a predominant component of rapeseed oils (e.g., *Brassica napus* up to 45%).

For reasons of health, the level of erucic acid in oils and fats intended for human consumption is limited by government regulation to a maximum of 2 and 5% of total fatty acids in the USA and EU, respectively. However, since erucic acid is the precursor of nervonic acid, higher erucic acid contents like in *Cynoglossum* species correlate with higher nervonic acid contents (up to 3.6%; Table 2). Nervonic acid, for instance, is considered crucial for biosynthesis of nerve cell myelin. VLC-MUFA-rich oils might therefore be relevant as nutraceuticals for the treatment of disorders involving demyelination, such as adrenoleukodystrophy and multiple sclerosis 29. Comparable amounts of these *n*-9 VLC-MUFA to the herein presented Cynoglossum species have previously been found in *C. divaricatum* 10. Interestingly, neither erucic acid nor nervonic acid were found in *C. officinale* and *C. nebrodens*e 16, despite comparable amounts of eicosenoic acid (5.1% vs. 5.5%; Table 2).

From the economic point of view, various parameters have to be taken into account (e.g., oil content and oil yield of seeds, growth period, cultivation conditions). In addition, the availability and sustainability of the new oil sources and for the use in human nutrition the existence of toxic compounds, for example pyrrolizidine alkaloids in Boraginaceae, should be investigated.

Apart from the diversity of seeds, the yielded oil content is dependent on oil extraction procedures and conditions, respectively (e.g., solvents: *n*-hexane, chloroform, petroleum ether and methods: Soxhlet extractor, supercritical fluid extraction 30). Compared to further analysed identical plant species we found similar oil contents for example in *Cynoglossum officinale* (15.6–19.4% 16 vs. 17.4%; Table 2), *Echium vulgare* (13.8–16.6% 16 vs. 15.3%; Table 2) and *Omphalodes linifolia* (16.5% 11 vs. 15.4%; Table 2). However, compared to the present oil contents both higher (e.g., *Cannabis sativa L*. cultivars 26.3–37.5% 31 vs. 18.6%; Table 2) and lower (e.g., *Cynoglossum officinale* 10.7% 11 vs. 15.6–19.4%; Table 2) oil contents have been reported.

In general, the oil content and the fatty acid profile can differ considerably between and even within one species, reflecting the influence of factors like vegetation, climate, degree of ripeness, management regimes, soil cultivations, testing site. In addition, minor genetic variations 12 and not least different methodical parameters (fatty acid transmethylation and GC conditions) could influence the fatty acid profile, too.

In conclusion, an important strategy to reduce the *n*-6/*n*-3 ratio in the diet is to increase its *n*-3 PUFA content. Considering the overfishing of the seas, the integration of *n*-3 PUFA-rich plant oils, particularly SDA-containing oils, into the diet could be a promising step forward towards ensuring the *n*-3 PUFA supply of an increasing world population. It is therefore assumed that screening of the fatty acid composition of less-researched plant seed oils may provide a nutritional benefit.

## Abbreviations

AA: arachidonic acid
ALA: α-linolenic acid
c: *cis*
DHA: docosahexaenoic acid
EPA: eicosapentaenoic acid
GLA: γ-linolenic acid
LC-PUFA: long-chain polyunsaturated fatty acids
LNA: linoleic acid
SDA: stearidonic acid
SFA: saturated fatty acids
TMSH: trimethylsulphonium hydroxide
